# An Interesting Letter

**Published:** 1871-05

**Authors:** 


### AN INTERESTING LETTER.

The following letter we take from the April number of that excellent medical monthly, the Journal of the Gynaecological Society of Boston. The positions taken are identically those announced in the previous numbers of the COMPANION. That they are correct and the only principles upon which the good men of the profession can stand and battle, no unprejudiced and intelligent member of the profession will question. The idea that men can have the hardihood to declare for themselves the right to affiliate with parties declared by our authorized bodies to be irregular or expelled therefrom, is preposterous, and amounts to puerile trifling with the good sense, and toying with the honor of the profession. Men may impose upon themselves so stupendous an absurdity, but they do it at the risk of being set down as illogical or worse, as attempting to imperiously override the laws and ethics of the profession. When they do the latter, sooner or later their schemes and plans will fall through and bring the perpetrators to grief. If in Georgia men will so far forget the true principles of the profession by the slogan cry of "my alma mater must be defended, right or wrong, at all hazards," then we tell them they have unconsciously, perhaps, signed the death-warrant of an institution requiring such sacrifice of principle to sustain it. It will inevitably perish under the withering simoon of professional wrath, and the last stage of such an institution will be worse than the first. So, too, with individuals. When a man, for whatever motive, deserts the principles of the profession, his days as a man worthy of recognition or entitled to prominence in the ranks of the true, are numbered. The finger of destiny, with the unfailing certainty of the doom which foreshadowed the overthrow of Belshazzer, will trace its sentence against him upon the tablets of the hearts and consciences of his professional brethren—"thou art weighed in the balances and art found wanting."

Time—the great restorer of principles lost or sacrificed—is the great logician to write the obituary of those who pander to error or bend the suple knee to wrong that "thrift may follow fawning."

"We have just received the Feb. No. of the Gynæcological Journal and desire to express our gratification. We trust we are actuated by no other motive than that of the good of the entire profession; but when any of its members are assailed simply because of their devotion and adhesion to the laws and principles of the profession, such an one should receive the support of all, from Maine to California. This principle must be established, or the profession will take no interest in our organization. It is our duty to hold up the hands of our brethren in Boston, as of any other part of the country. When individuals abroad refuse to sustain those who are waging a good professional fight at home, because of remoteness or from any other cause, the profession is damaged, and our Association becomes not only useless but undesirable. No individual can isolate himself or play the part of a neutral, when vital principles are at stake, simply because his private interests are not involved. In other words, he is aiding to sacrifice his professional principles, or ignore them, when others at a distanee are suffering mental and physical torture to maintain them. This is the essence of selfishness and baseness. We feel that every member of the profession should be taught to view this principle in this light, if anything good is ever to be accomplished.

"Those who belong to the State and the National Organizations should not be permitted to enjoy the privileges which these organizations confer upon them when, at home, they recognize and affiliate with quacks, irregulars and expelled members from these organizations. If such men can enjoy such privileges, why the necessity of disbanding a society, as in the case of the Berks County Society, published in your last number? If the "notorious abortionist" is to be consulted with by men who can be represented in the Association, why disband one society in order to form another? Why,—if the abortionist loses nothing, but is taken by the hand, encouraged and supported by members of State and National Associations, at home? And if his *personal friends* are to lose nothing by recognizing the abortionist, but are to be esteemed good and loyal members of the associations, why the necessity of having any law or association at all? These are the points and principles which are to be established before the profession can maintain its dignity, or expect to command the respect of its members, or of the public.

"Now we know that you are not only qualified, but willing, to advocate these principles. We all feel the importance of doing so. The American Medical Association should, at its next meeting, forever set at rest this subject of affiliation. In order to aid this desirable result, we must lay down the law and show what must be done by each individual member, in order to maintain his connection with our societies and the Association. An individual who is not a member of any society, in order to membership in the National Association, should be a man of high character, and be vouched for by members present, that he has never been expelled from any local or State society. No one who is under sentence of expulsion or suspension from any State or local society of which he may have been a member, should be received until he has been properly restored. No college or institution should be represented which is not in good standing at home, or has been refused recognition by the State Association; and no society should be permitted representation who allows its members to affiliate with irregulars or members expelled either for moral or ethical violations. This will establish the course you have taken and that of the Berks County Society, and if adopted by the profession, it will bring harmony and order in our ranks. If we do not do this work, there will be trouble, in May, in California."

